# Organic waste as a sustainable feedstock for platform chemicals

**DOI:** 10.1039/c7fd00070g

**Published:** 2017-02-22

**Authors:** M. Coma, E. Martinez-Hernandez, F. Abeln, S. Raikova, J. Donnelly, T. C. Arnot, M. J. Allen, D. D. Hong, C. J. Chuck

**Affiliations:** a Centre for Sustainable Chemical Technologies (CSCT) , University of Bath , Claverton Down , Bath , BA2 7AY , UK . Email: m.coma@bath.ac.uk; b Department of Chemical Engineering , University of Bath , Bath , BA2 7AY , UK; c Centre for Doctoral Training in Sustainable Chemical Technologies , Department of Chemical Engineering , University of Bath , Claverton Down , Bath , BA2 7AY , UK; d Plymouth Marine Laboratory , Prospect Place, The Hoe , Plymouth PL1 3DH , UK; e Algal Biotechnology Department , Institute of Biotechnology , Vietnam Academy of Science and Technology , 18 Hoang Quoc Viet Str., Cau Giay , Hanoi , Vietnam; f Water Innovation & Research Centre , University of Bath , Claverton Down , Bath , BA2 7AY , UK

## Abstract

Biorefineries have been established since the 1980s for biofuel production, and there has been a switch lately from first to second generation feedstocks in order to avoid the food *versus* fuel dilemma. To a lesser extent, many opportunities have been investigated for producing chemicals from biomass using by-products of the present biorefineries, simple waste streams. Current facilities apply intensive pre-treatments to deal with single substrate types such as carbohydrates. However, most organic streams such as municipal solid waste or algal blooms present a high complexity and variable mixture of molecules, which makes specific compound production and separation difficult. Here we focus on flexible anaerobic fermentation and hydrothermal processes that can treat complex biomass as a whole to obtain a range of products within an integrated biorefinery concept.

## 1. Introduction

Two main challenges for our society are the depletion of fossil resources and increasing waste generation. In order to reduce the dependence on oil but also mitigate climate change in the transport and chemical sectors, alternative production chains are necessary.^1^ This involves a shift towards renewable resources, which are not finite and can be easily regenerated. While the energy economy can be based on various alternative raw materials (wind, sun, water, biomass, nuclear fission and fusion), the material economy of substances mainly depends on biomass, in particular plant biomass. Therefore, biorefineries as bioresource-converting systems, analogous to petroleum-based refineries, will be the key for access to the bioeconomy: an integrated production of biobased products (food, feed, chemicals and materials) and bioenergy (fuels).^2^

**Fig. 1:**
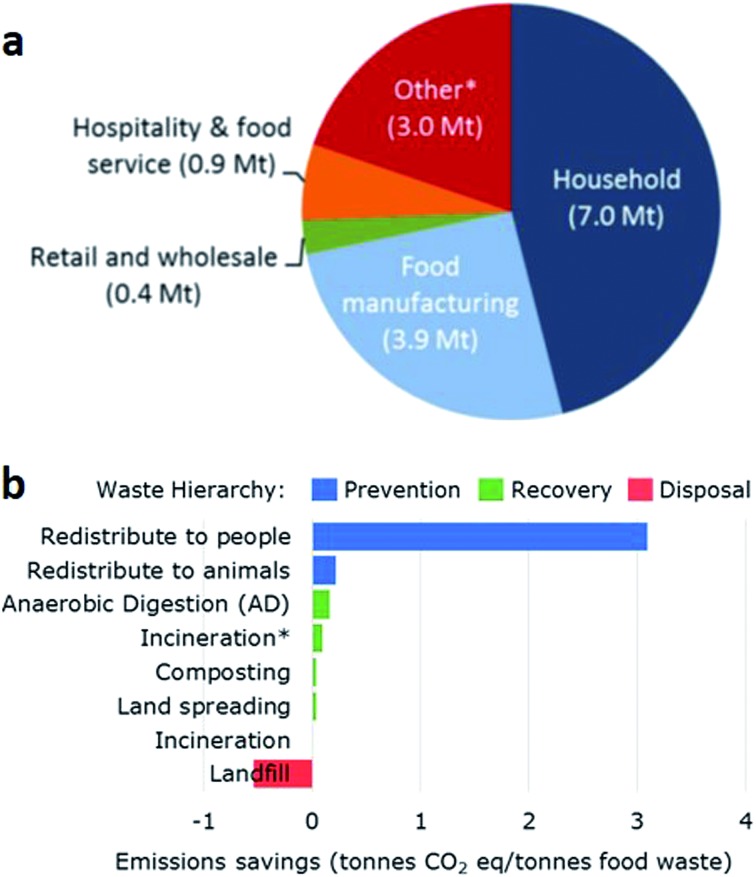
(a) Sources of organic waste in the UK. *Includes pre-factory waste. (b) Emission savings per tonne of organic waste depending on its management. *Includes energy recovery.^5^

Waste generation is the second major challenge for our society. Nearly 50% of the average composition of global waste, 3 M tons per day, is organic material. This accounts for the main source of greenhouse gases (GHG), and it includes household, food manufacturing and pre-factory wastes, the rest being paper, plastic, glass, metal and others (Fig. 1).^3^ These waste streams contain various compounds, most of which have untapped energetic or economic value. Current waste management practices in decreasing order of added value for organic waste include: animal feed, composting, incineration and landfill. Although a number of facilities direct their waste toward land spreading, these facilities only represent between 26% and 46% of the organic waste and most of it is still disposed of in landfills.^4^

### 1.1. Towards a more sustainable biorefinery

Biofuels such as bioethanol and biodiesel produced from seeds, grains and sugar (the so-called first generation (1G) biofuels) have exponentially increased in use since the 1980s due to their easy applicability within existent engines without modifications, their renewability, their biodegradability, their lower emission generation and the ability to increase the security of the supply and provide a steady income to farmers.^6^ However, energy carriers produced from crops have caused inflation in food prices and led to the food *versus* fuel crisis. Hence, the production of bioenergy from alternative sources such as agricultural and domestic organic wastes, the substrates for second generation (2G) biofuels which are mainly composed of lignocellulosic biomass, has now provided a positive shift towards green energy production.^7^ 2G biofuels generated from non-crop feedstocks release the pressure on the food market; however, there is concern over competing land use or required land use changes. Therefore, third generation (3G) biofuels have been derived from past agricultural substrates, waste vegetable oils, microbes or microalgae as a viable alternative energy resource.^6^ The biorefinery concept embraces a wide range of technologies to separate biomass resources into their building blocks (carbohydrates, proteins, triglycerides and others) which can be converted not only to biofuels, but also to value added products and chemicals.

Most of the existing biofuels and biochemicals are currently generated in single production chains and not within a biorefinery concept, and thus their exploitation is thereby limited.^1^ Modern biotechnology focusses on biofuels. Biogas production uses a resilient ecosystem of diverse microorganisms to co-convert multiple organics into methane. Bioethanol production is only possible with single bio-based substrates and single yeast strains, and its efficiency strongly depends on the bioavailability of carbohydrates. In general, these processes convert only a fraction of organics into biofuel and other outcomes are low value co-products or waste. To overcome this drawback, waste streams should be managed *via* a biorefinery system which integrates technologies flexibly and where all product outcomes are considered. The design and development of multi-purpose biorefineries that generate a variety of products as a consequence of integrated, sequential, non-competitive processes is considered a strategic way to reach this goal.^8^

### 1.2. Organic waste as a feedstock

Organic waste streams are a sustainable alternative to fossil-based resources as they do not compete directly with food crops. ‘Waste’ covers any organic material apart from the primary material for which the plants were originally grown (*e.g.* corn stover from maize), but it also applies to any biomass-derived by-product for which supply greatly exceeds demand (*e.g.* glycerol from biodiesel). Nearly all waste streams currently have some value, for instance agricultural waste is used as a soil improver in the fields, but the future looks toward obtaining a higher value from them. Most biorefineries utilise the available feedstocks without upstream concerns, which are grouped into different categories such as lignin, carbohydrates, proteins and triglycerides (from fat and oils). Lignin, from woody biomass, can be used as a fuel but it is also the only large volume renewable that comprises aromatics. Bulk chemicals can be obtained after hydrolysis of the carbohydrate residues to their monomers to obtain, among others, bioethanol, butanol and lactic acid from fermentation, or furfural and 5-hydroxymethyl-furfural produced from acid-catalysed dehydration of pentoses. Proteins residues (*e.g.* distillers grains) are valorised as animal feed, but the ideal scenario would be to isolate the non-essential amino acids as chemical feedstocks.^1^,^9^

Rapid industrialisation across the developing world has led to a number of adverse effects on the environment. The severe dumping of plastics into water courses has fouled our oceans, rivers, lakes and estuaries; while excessive nutrient run-off from intense agricultural activity has prompted unchecked and persistent micro- and macroalgal (seaweed) blooms, which are potential feedstocks for 3G biorefineries. Harmful algal blooms (HABs) can produce anoxic zones, kill wildlife and produce toxic compounds responsible for death, illness and/or a direct restriction of commercial activities such as fishing and tourism.^10^ Whilst they are relatively chemically inert, there is an increasing body of evidence for the detrimental impact of plastics on aquatic wildlife and trophic food webs, in addition to the obvious impact of detritus on the aesthetics of the environment.^11^ It is of particular relevance that these vastly different anthropogenic pollutants often become entwined with the complex organic matrix associated with HABs.

Sustainable feedstocks can include energy crops grown on marginal land, agricultural and forestry residues, municipal solid waste, and other novel feedstocks such as algae and other aquatic plants and microbial biomass.^12^ However, the variability of the quantity and composition reduces the technological and economic feasibility of potential value conversion processes. The types of biomass sources not falling into the categories mentioned above, but commonly used in biorefineries, are the organic fraction of Municipal Solid Waste (MSW), manure, sewage sludge, wild fruits and crops, proteins and residues from fresh fruit and vegetables or food waste (FW). The physical and chemical characteristics of these wide spectrum biomass resources vary largely and, therefore, they are more suited for systems that can recover the potential of the organics as a whole. Examples might be anaerobic digestion (AD) to produce biogas or hydrothermal systems to produce a crude oil substitute.

### 1.3. Biologically based conversions for complex biomass

AD is based on a mixed microbial biotechnology (MCB) that originated from the waste treatment field. Compared with pure culture-based industrial biotechnology, MCB does not require sterilisation, has a high adaptive capacity, can use mixed substrates thanks to microbial diversity and it is possible to operate it as a continuous process.^13^ The biogas produced during AD is the final product of a long chain of reactions, including hydrolysis of polymers to monomers and oligomers, oxidation of these products during primary fermentation generating volatile fatty acids (VFAs), lactate and ethanol, as well as hydrogen and carbon dioxide, and a final secondary fermentation. All these biochemical reactions are enclosed within the carboxylate platform in which carboxylates are the intermediates or final targets for conversion of biomass to chemicals or biofuel.^14^

Hydrogen generated during acidogenesis (primary fermentation) has the highest energy content per unit weight of any known fuel (143 GJ tonne^–1^) and is the only fuel that does not contain any carbon. In comparison to the combustion of methane, which is generated by AD, hydrogen combustion is considered a cleaner technology as it does not involve carbon dioxide.^7^ However, methane can be generated alongside the hydrogen in low quantities either by typical acetoclastic methanogens (from acetate) or by hydrogenotrophic organisms (from H_2_ and CO_2_). A mixture of hydrogen and methane is known as biohythane (46–57% H_2_, 43–54% CH_4_, 0.4% CO_2_) and it is a perfect fuel owing to its cleaner nature than methane, high fuel efficiency, improved heat efficiency and ability to make engines easy to ignite with less input energy. The value of these technologies does not rely only on the biogas obtained anaerobically, but also on the other biobased products present in the fermentation broth which may have a high commercial value in the market.^7^,^15^

The different types of products obtained within the carboxylate platform will depend on the substrate composition and operational conditions, which will control the syntrophy between different organisms and determine the final microbial community.^16^,^17^ In all microbial fermentations where organic carbon is both the electron donor and acceptor for the redox reactions, methane presents the lowest Gibbs energy change and thus a homogeneous end-product will be generated irrespective of the substrate.^18^ Thus to switch from biogas to biochemical production in MCB, methane production must be inhibited by working at suboptimal AD conditions. Preliminary studies are required of diverse organic streams as composition variability will modify the synergy between organisms, and thus the biological reactions and final product.

### 1.4. Hydrothermal based conversions for complex biomass

Algal biomass and plastics each represent different, yet significant, opportunities and problems from a remediation perspective. Algal biomass requires extensive drying to obtain a suitable feedstock for processing, rendering direct combustion or pyrolysis routes, normally used for plastic waste, uneconomic.^19^ However, suitable conversion technologies for wet biomass, such as anaerobic digestion (AD) and fermentation, are not able to convert the energetically rich plastics. Hydrothermal liquefaction (HTL) offers an interesting opportunity for simultaneous processing of heterogeneous organic material. In HTL, biomass is processed wet, with solid loadings of 5–20%, at 280–350 °C under pressures of up to 180 bar. HTL results in four product phases, including a bio-crude oil that can be processed into fuels and chemicals similarly to crude oil, an aqueous phase containing nitrate and phosphate based micronutrients, CO_2_, and a solid residue containing the inorganic elements and further carbon.^20^

### 1.5. A multi-product integrated biorefinery for organic waste

Biorefineries must be developed using process design, technology integration, and analysis of the sustainability and economics. We are working towards a biorefinery that will produce different products from various sustainable feedstocks by integrating upstream, processing and downstream stages, taking into account both biological and thermo-chemical technologies and their integration for maximum recovery. Various wild yeast strains have been applied to either produce lipids from depolymerised lignocellulose^21^ or microbial palm oil substitutes.^22^ Bacterial communities have been investigated for the production of antimicrobials, using the intermediate products of anaerobic digestion and bioethanol obtained from side-streams.^16^,^23^ Fuel precursors as a bio-oil have been obtained from physical treatment of algal biomass.^24^ Although recently advances in bio-based feedstock processing have been achieved, a holistic perspective is still required to allow efficient technology integration, which is justified by sustainability and economic analysis. To achieve this, previous knowledge of the substrate behaviour in each technology is required. Evaluation of the processes and their integration can be achieved thanks to system modelling. Process system analysis tools enable integration of multi-step processes for maximisation of the energy and resource recovery efficiency, and mitigation of the emissions, waste and cost,^25^ achieving an interdisciplinary approach for sustainable feedstock valorisation. In this work, we evaluate two separate biological and thermochemical systems for organic waste streams, the latter is used with plastics as the main impurities in the feedstock. The final aim was to unravel the energy and chemical potential of different substrate compositions to be applied to an integrated biorefinery concept.

## 2. Results and discussion

### 2.1. Biological conversion of food and liquid waste to biogas and chemicals

An anaerobic digestion process is applied for complex biomass conversion into methane. Its microbiome containing hydrolisers, fermenters and methanogenic organisms in syntrophy allows the oxidation of variable substrates to a single product. High concentrations of VFAs or a low pH cause inhibition of the methanogenesis. Limiting methane production increases the possibility of recovering chemicals and alternative fuels from these systems. Under conditions of overloading and in the presence of inhibitors (*e.g.* free ammonia, high salts), the methanogenic activity cannot remove hydrogen and volatile organic acids as quickly as they are produced. The result is accumulation of acids, depletion of the buffering capacity and depression of the pH to levels that, sometimes, also inhibit other fermentation processes.^26^ Several fermentation processes for carboxylic acids have been pursued at a low pH (*i.e.* below or close to the lowest p*K*_a_) although higher titers were achieved at neutral pH, which in carboxylic acid production is between pH 6–8.^27^ At a pH of around 6, short carboxylates can be recovered as bulk chemicals, and further conversion of them is possible to medium-chain VFAs with a higher value^28^ or hydrogen can be simultaneously generated as an alternative fuel. Therefore, evaluation of the fermentation products and biomethane potential by adjusting the load and pH within MCB allows determination of the maximum energy and chemical production and composition. Here we investigated the influence of the food to microorganisms ratio during anaerobic conversion of food waste (FW) and determined the acidification potential (AP) and biochemical methane potential (BMP) of various organic substrates.

#### 2.1.1. Influence of inoculum concentration

**Fig. 2:**
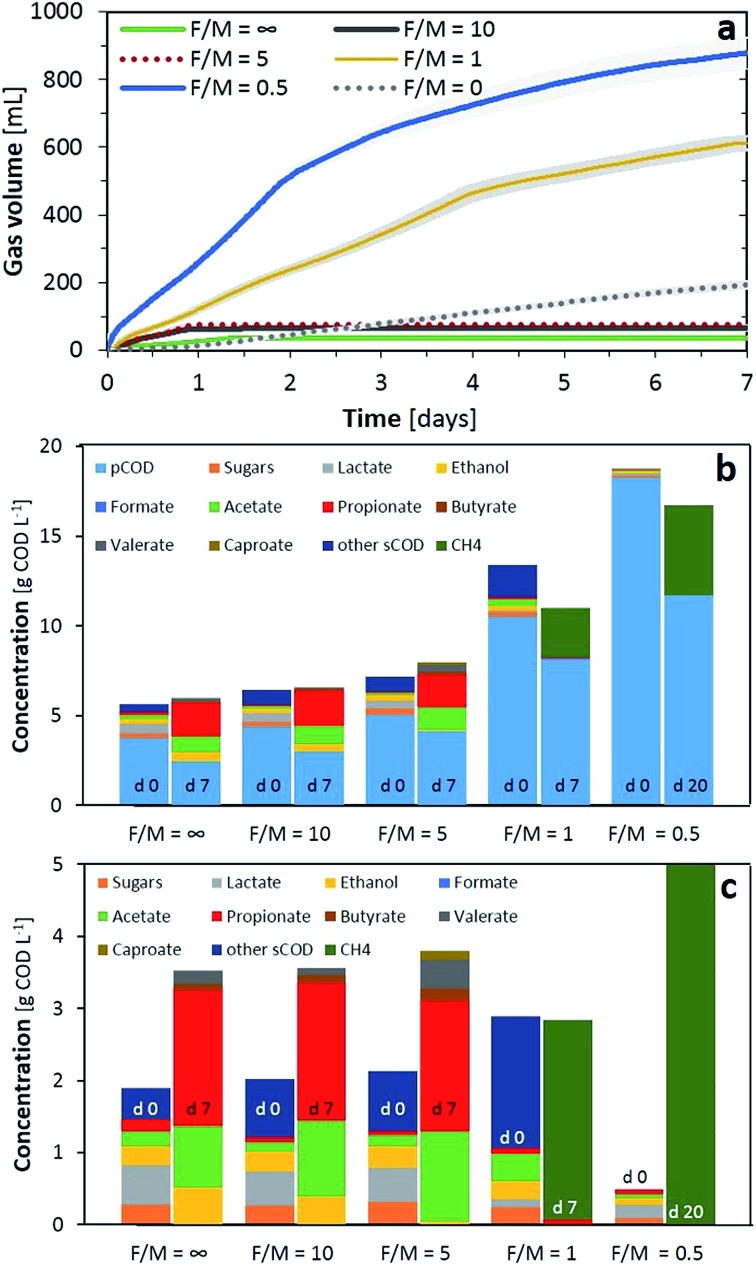
(a) Gas production during the first few days of anaerobic fermentation; (b) total COD composition at the start and end of each experiment; (c) soluble COD composition at the start and end of each experiment. Tests were carried out at various food to microorganism ratios (F/M, g COD g^–1^ VS). F/M = ∞ refers to only substrate (5 g COD L^–1^) while F/M = 0 to only inocula (10 g VS L^–1^).

A substrate concentration as chemical oxygen demand (COD) of 5 g COD L^–1^ of FW was mixed with different concentrations of an AD mixed microbial inoculum to obtain various food to microorganisms (F/M) ratios of ∞, 10, 5, 1 and 0.5, corresponding to a volatile solids (VS) concentration of 0, 0.5, 1, 5 and 10 g VS L^–1^, respectively. A F/M ratio of 0 was used as an inoculum control with 10 g VS L^–1^ and no substrate. Biogas production was only significant for F/M ratios of 1 and 0.5, steadily increasing at a rate of 112 and 215 mL d^–1^, respectively, during the seven days of incubation (Fig. 2a). As for the rest of the conditions, the pH decreased below 6 during the first day of fermentation (data not shown). About 100 mL of biogas was produced for F/M ratios of 5 and 10 during the first day, probably H_2_ from acidification, and the rates decreased to zero after that day. For F/M = 0, biogas (60% CH_4_) was produced at a rate of 27 mL d^–1^, a product of conversion of the soluble substances and auto-digestion of the sludge. Regarding the total COD supplied in the tests, increasing the inoculum concentration not only increased the particulate organics (pCOD, Fig. 2b) but also other soluble organics inherent in the sludge. For the fermentations with low inoculum, the total COD was similar at the start and end of the test, while this balance was not present for F/M ratios of 1 and 0.5 probably due to losses through H_2_ and CO_2_, which were not accounted during the experiment.

In terms of soluble COD, using the substrate alone or F/M ratios of 10 and 5 solubilised a fraction of the pCOD as can be observed from the increase in soluble substances at the end of the experiment. Sugars and lactic acid were primarily converted into propionic acid (51–54%), acetic acid (24–36%) and ethanol (2–15%) with traces of butyric (2.5–5%) and valeric (3–11%) acids. Not surprisingly, the conditions which resulted in the lowest ethanol (F/M of 5), produced 3.5% of caproic acid. This is in line with a possible chain elongation of VFAs using common organisms present in AD (*e.g. Clostridium*), which can reverse the β-oxidation reaction under the reductive conditions supplied by hydrogen and ethanol as the electron donor.^29^ Therefore, a concentration of 1 g VS L^–1^ (F/M of 5) was chosen as the optimal concentration to evaluate the acidification potential (AP) of the substrates, as minimal carbon losses were detected from the total COD while this inoculum concentration even allowed biological upgrade of the short VFAs into more valuable chemicals such as caproic acid. For a F/M ratio of 1, the main loses were detected as being due to biogas production, while optimal methane production was obtained with a F/M of 0.5 in line with values in the literature.^30^

#### 2.1.2. Effect of organic loading

**Fig. 3:**
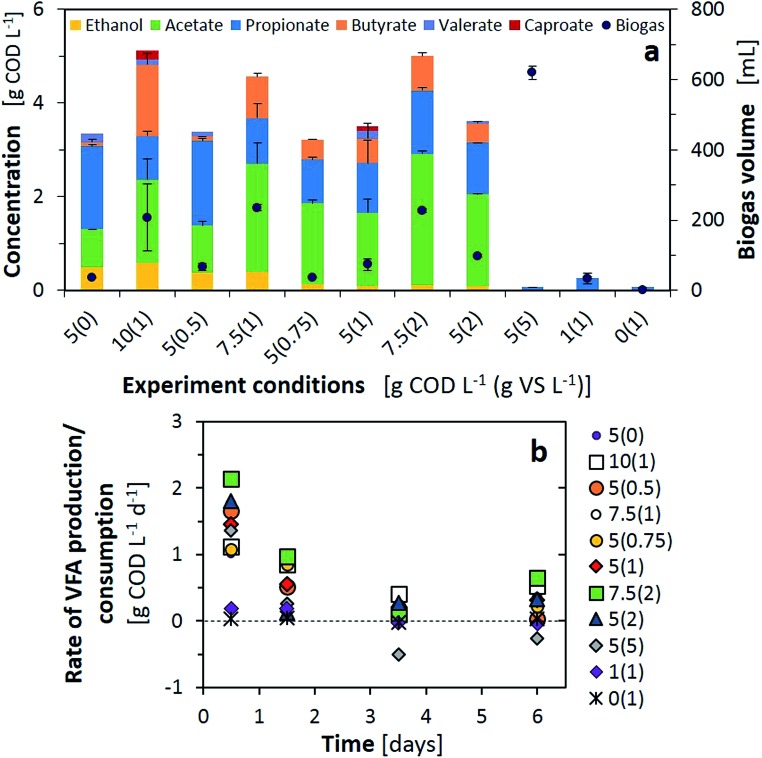
(a) VFA (left) and biogas (right) production and composition using various 7-day fermentation combinations; (b) rate of VFA production or consumption during the 7-day fermentation tests with various loads and F/M ratios. Experimental conditions indicated are the organics concentration from FW to inoculum [g COD L^–1^ (g VS L^–1^)].

A F/M ratio of 5 was found to be optimal for the AP when comparing the inoculum concentrations. However, microbes inherent in the feedstock when dealing with organic wastes also provide hydrolytic and acidogenic activity when incubated for fermentation purposes (F/M = ∞, Fig. 2). Therefore, the quantity of organics, independent of the F/M ratio provided by the inoculum, may also increase the microbial community and modify the AP outcome when dealing with waste streams. Fig. 3 presents various combinations of organic loading rates and the inoculum, all with F/M ratios equal to or above 1, with the exception of the control inoculum experiment (this time with 1 g VS L^–1^).

As initial AP tests indicated, F/M ratios of 1 did not lead to fermentation products; at minimum loadings nearly no VFAs, ethanol or biogas were produced, while at higher loadings of 5 g COD L^–1^ all the organics were converted to biogas (Fig. 3a). These results were also confirmed by the minimal VFA production rate or even negative rate from day 3, indicating consumption of the VFAs in methane production (Fig. 3b). From a F/M ratio of 2 and above, all the tests produced similar or higher concentrations than the substrate alone (F/M = ∞, 5(0)). We observed that with similar F/M ratios, an increase of inocula boosted the total VFA production and reduced the presence of propionate by 10–30% in the final composition (*e.g.* 5(0.5) to 10(1) or 5(0.75) to 7.5(1)). Ethanol predominated after the 7-day fermentation for the tests with a maximum organic loading over the inoculum; however, an ethanol peak of 0.7 g COD L^–1^ was observed at day one and depleted afterwards for the tests with 5(0.75), 5(1) and 5(2). Lactic acid was consumed only, from the substrate, except for the test with 10(1) wherein the lactic acid concentration peaked at 0.5 g COD L^–1^ after one day and was depleted during consecutive days. Ethanol and lactate consumption, as electron donors for chain elongation reactions, should have increased the longer chain VFAs. This situation was only correlated with results from 10(1) and 5(1), with the production of caproic acid. Finally, the tests with higher inoculum concentrations but a F/M ratio over 2 provided comparable or higher VFA concentrations than the rest and an improved VFA production rate for the first two days of fermentation as well as during the last day (Fig. 3b). Although higher loading rates were providing an improved VFA concentration, the conversion yield (*Y*_VFA_), which stands for the conversion of total COD to VFAs, was reduced below 50% in such cases. A maximum yield of 70% was obtained with 5(1), in line with conversions from the literature obtained for sugar based substrates converted to methane or to VFAs.^17^,^31^ While similar values for the VFA production rate and *Y*_VFA_ were obtained for 7.5(2) and 5(2), ethanol production, and thus possible further valorisation to long chain fatty acids, was limited. Therefore, optimal test conditions of F/M = 5 with 1 g VS L^–1^ were adopted for further AP tests.

#### 2.1.3. Effect of substrate composition on the VFA and methane production

**Fig. 4:**
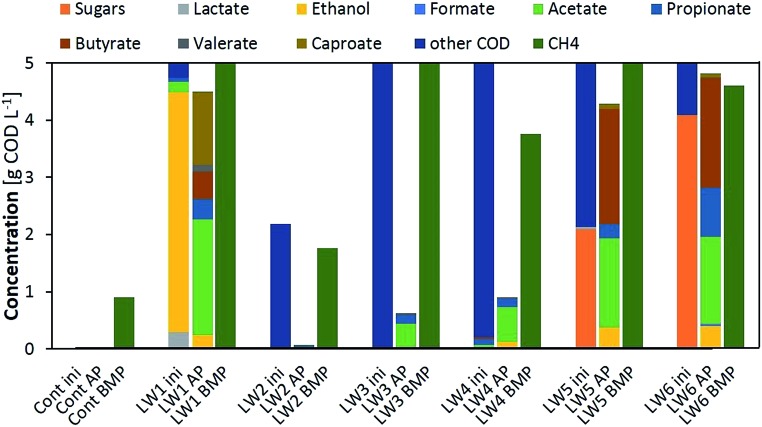
VFA and methane production, and the obtained compositions, from 7-day acidification potential (AP) tests and a 30 day biochemical methane potential (BMP) test using 6 different liquid wastes. Cont stands for control while ini stands for initial composition.

Optimised AP and BMP tests were carried out for six different liquid wastes (LW1–LW6) to determine the conversion potential to VFAs or methane. Fig. 4 presents the initial composition of the added substrate (5 g COD L^–1^, except for LW2 which was a diluted substrate) and the end composition for both the AP and BMP tests.

LW2 presented a nearly non-detectable VFA production for the 7-day AP test but converted nearly all its initial COD into methane during the BMP test. This was due to an extremely slow hydrolysis process confirmed by a constant of 0.05 d^–1^ (Table 1). Thus a longer residence time of 30 days allowed substrate degradation to occur. Similar profiles with low amounts of VFA products for the AP and high yields for the BMP tests were observed for LW3 and 4, again with lower hydrolysis constants that would limit further fermentation steps.

**Table 1:** pH variation, efficiency and kinetic parameters of the AP and BMP tests for the different substrates tested

	Acidification potential (AP)				Biochemical methane potential (BMP)			
	ΔpH	DA g COD_VFA_ g^–1^ COD_fed_	*Y*_VFA_%	*R* 0 VFA g COD_VFA_ L^–1^ d^–1^	ΔpH	SMP mL CH_4_ g^–1^ COD_fed_	*Y*_BMP_%	*K*_h_ d^–1^
FW	–2.00	0.59	102	1.45	–0.3	377	108	0.36
LW1	–2.71	0.78	94	1.16	–0.2	407	116	0.64
LW2	0.29	0.00	1	–0.03	0.05	348	100	0.05
LW3	–1.57	0.12	16	0.26	–0.65	462	132	0.24
LW4	–0.61	0.11	37	0.26	–0.4	264	75	0.46
LW5	–3.25	0.74	81	2.07	–0.5	373	107	0.56
LW6	–3.29	0.82	87	1.89	–0.5	320	92	0.57

Substrates with a high proportion of ethanol or sugars in their initial composition (LW1, LW5 and LW6) presented the highest VFA production as well as *Y*_VFA_, which was over 90% for the substrate mainly composed of ethanol. Ethanol oxidation to acetate did not only improve the increase in VFAs, but provided the required energy to initiate chain elongation reactions^28^ and, thus, generate butyrate from acetate and furthermore caproate from butyrate. Nearly no biogas, only 18 mL, was produced from LW2, consisting of 4% CO_2_ and the rest gases other than methane, supposedly hydrogen. This would have also enhanced the reductive conditions required for chain elongation. In the case where sugars were present within the initial substrate, the monomers had to undergo acidification to produce the required ethanol and acetate, accompanied with maximal biogas production other than methane (25% CO_2_, 75% H_2_), therefore chain elongation was only extended to butyrate during the 7-day fermentation.

Kinetic parameters obtained from both the AP and BMP tests corroborated that VFA production relies on the hydrolytic capacity of the substrate as well as the activity of the microbiome. Lower VFA rates were obtained for those substrates with lower hydrolysis constants (LW2–4). In such cases, the substrates should be pretreated to enhance the initial step of fermentation or only applied for biomethane production with larger retention times. For sugar based substrates, even relatively low hydrolysis constants would allow a high recovery of VFAs together with a potential for biohydrogen recovery. These systems also presented the highest pH decrease during fermentation, thus potential inhibitions should be taken into account. Finally, ethanol based substrates can also be considered for VFA recovery with the added value of production of longer chain VFAs.

### 2.2. Hydrothermal co-liquefaction (HTL) of algal and plastic wastes

**Fig. 5:**
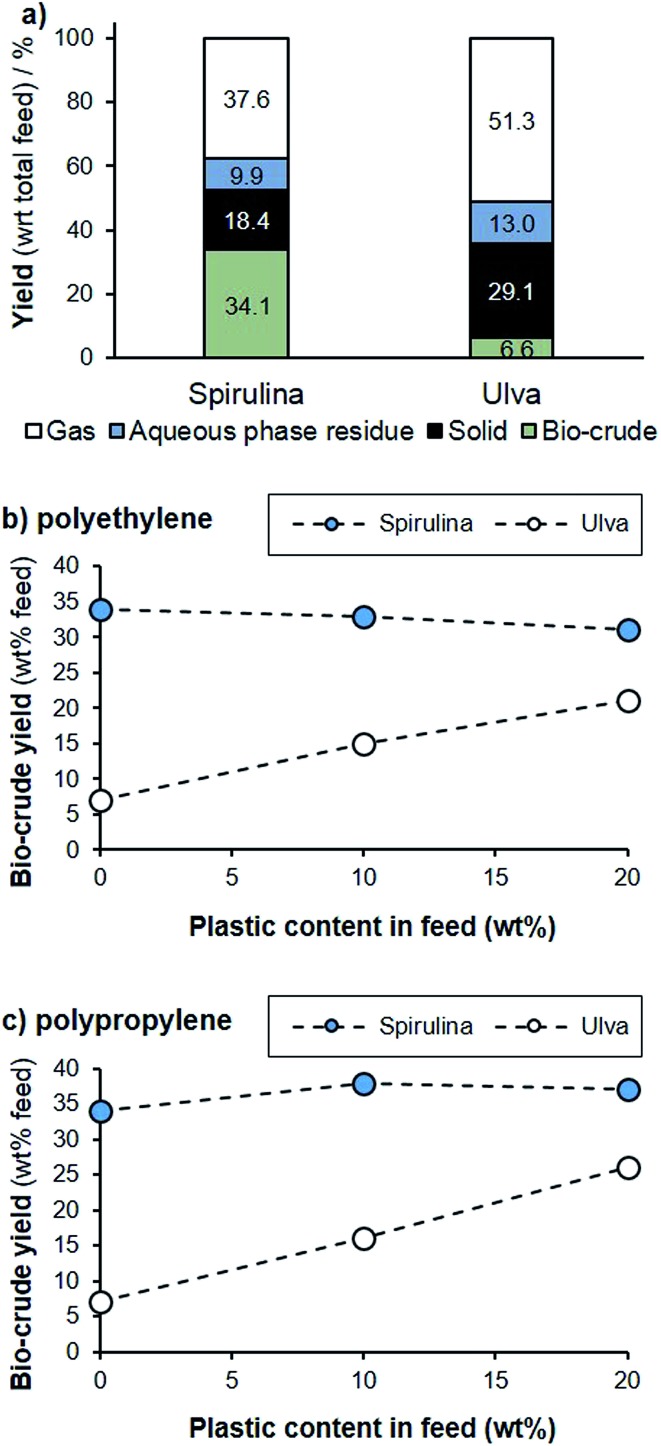
HTL of Spirulina and *Ulva* spp. (310 °C, 60 min): (a) product mass balance of the biomass; (b) bio-crude yield with increasing PE content; (c) bio-crude yield with increasing PP content.

While the hydrothermal co-liquefaction of plastics in water has been examined with lignocellulose,^32^ no reports detail the effect of plastics on the HTL of micro- or macroalgae. To assess this application, we studied the microalgal species *Arthrospira platensis* (Spirulina) and the macroalga *Ulva* spp. (Ulva), both of which are common and problematic bloom formers in areas such as Vietnam. All samples were liquefied at 310 °C over 60 minutes. Under these conditions, an oil yield of 34% was obtained for Spirulina, while the liquefaction of Ulva produced only 7% biocrude (Fig. 5a). While this is substantially lower than with Spirulina, it is common for macroalgae to contain a higher polysaccharide and ash content, and correspondingly have lower oil yields. For example, other members of this genus have been reported to provide oil yields of 18–32% under similar conditions.^33^,^34^

Plastics such as polyethylene (PE) and polypropylene (PP) are more thermally stable than the algal biomass, and under the conditions tested both plastics failed to degrade when processed separately to the biomass. However, on co-liquefaction both PE and PP demonstrated significant degradation. For example, with addition of both PE and PP, the overall oil yield remains approximately stable for Spirulina, despite proportionally less biomass being present in the reaction mixture, where the addition of both plastics increased the bio-crude yield dramatically for Ulva. Presumably, as the biomass begins to degrade, these secondary products impact on the thermal stability of the polymers, which then react and decompose. The hydrocarbon polymer subsequently becomes a hydrogen donor, and this hydrogen can stabilise radicals formed during biomass decomposition and prevent recondensation to solid residues. This effect has been observed from co-liquefaction of lignocellulose with HDPE, for example.^32^

**Fig. 6:**
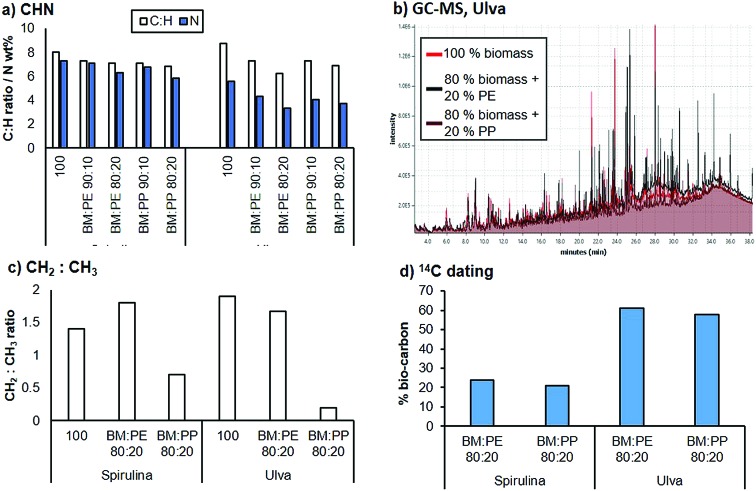
Analysis of the bio-crude fractions: (a) the carbon : hydrogen ratio and nitrogen content of the bio-crude with a varied plastic content; (b) GC analysis of the bio-crude derived from Ulva; (c) the ratio of CH_2_ to CH_3_ moieties estimated from the 1H NMR; (d) % fossil carbon in the bio-crude.

The bio-crude fraction was analysed using elemental analysis (Fig. 6a). On addition of PE or PP to the Spirulina biomass, the C : H ratio decreased compared to the bio-crude from the pure Spirulina. This effect was even more pronounced at higher plastic loadings. A similar trend was also observed for the bio-crude produced from Ulva. The N content was also reduced in the bio-crude fractions with as little as 3.3% being observed. Nitrogen tends to be present in aromatic heterocycles and as such requires extensive hydrotreatment for removal from the resulting crude upon chemical upgrading. Bio-oils containing a lower nitrogen content are therefore significantly easier to process.

GC-MS analysis of the bio-crude fractions demonstrated that the majority of the lighter fraction was composed of aromatic compounds, fatty acids and nitrogen containing heterocycles. While this did not change substantially for the co-liquefaction products, an increasing hydrocarbon content (C_10_–C_22_) was observed with the addition of PP and PE. For the liquefaction of Ulva, a significant proportion of additional saturated hydrocarbons were observed around the C_20_ range for both PP and PE liquefaction (Fig. 6b). This strongly suggests that the plastic is partially fragmenting and partitioning into the bio-crude fraction. ^1^H NMR analysis of the biocrudes demonstrated that the crude is relatively similar when PE is introduced, with approximately the same CH_2_ : CH_3_ ratios. However, following addition of PP, a larger proportion of CH_3_ groups were present, suggesting either deposition of the PP polymer chain or the production of shorter chain moieties from the biomass. To estimate the total carbon content from the biomass, ^14^C dating of the bio-crudes was undertaken, and it was demonstrated that between approximately 21–61% of the carbon in the bio-crude came from the biomass source, for the 20% polymer loadings. This equates to approximately 10–15% of the available carbon in the Spirulina biomass depositing into the bio-oil and between 45–55% of the carbon available in Ulva depositing into the bio-oil.

**Fig. 7:**
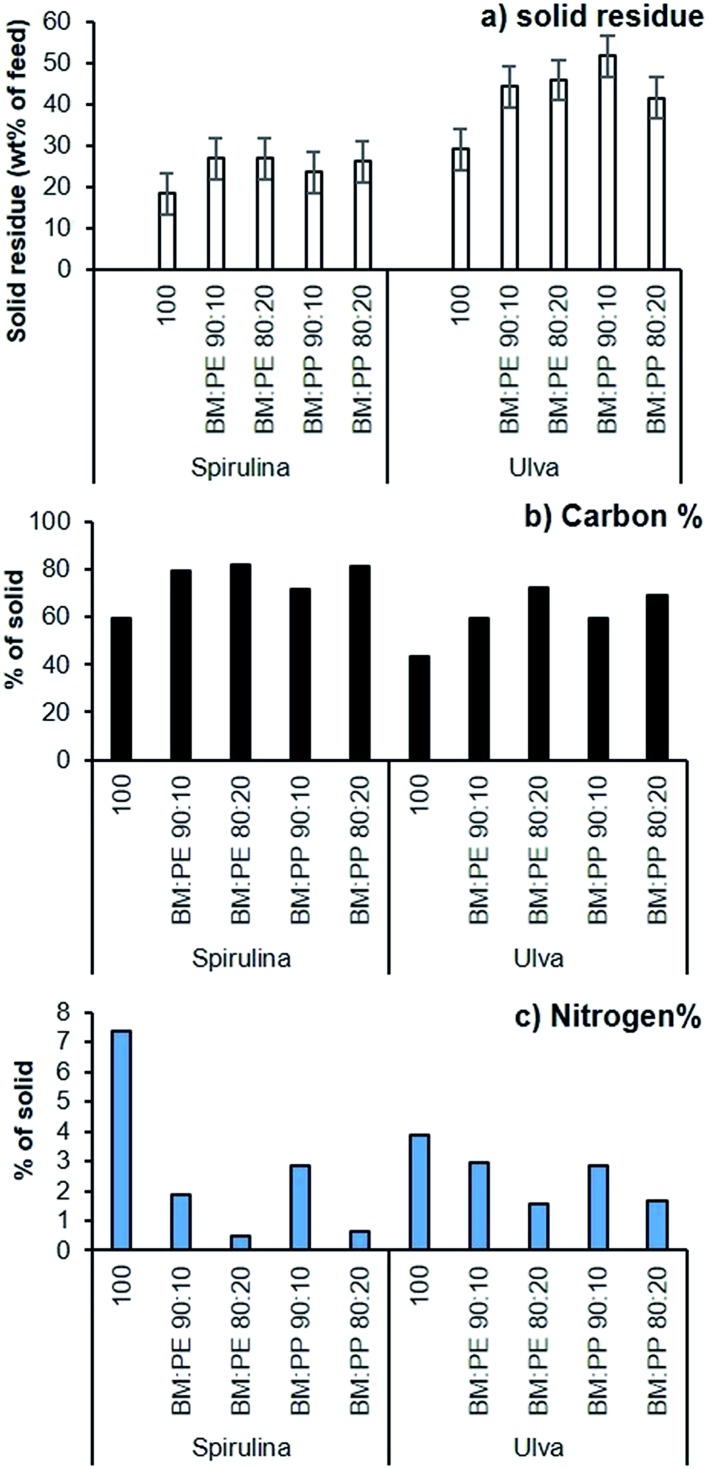
Analysis of the solid residues from the HTL of Spirulina and Ulva (310 °C, 60 min): (a) solid residue yield with increasing plastic loading; (b) carbon % from the elemental analysis; (c) wt% nitrogen.

The solid residue fraction was also increased by the addition of plastic, with higher loadings of plastic producing higher yields for Spirulina and Ulva (Fig. 7a), though this was far more pronounced for the macroalgae. The additional solid residues showed an increased carbon and decreased nitrogen content (Fig. 7b and c), both factors suggesting that some of the plastic waste was depositing in this phase, with more plastic available to deposit with the Ulva sample due to lower deposition in the bio-oil.

This exciting preliminary study demonstrates that the co-liquefaction of algae and plastics has significant potential for remediation of complex organic pollutants. Indeed, the additional plastics result in higher conversions to bio-crude, which was generally of a higher quality than the biocrude produced from algal biomass alone.

### 2.3. Organic waste biorefinery system integration

A biorefinery is a facility for the sustainable conversion of biomass and waste feedstocks, through the integration of physical, chemical, biochemical and thermochemical processes, into multiple products.^35^ The analogy to today’s crude oil refineries suggests that adoption of process system engineering principles, such as feedstock fractionation, multiple product portfolios, process flexibility and process integration, should be applied for biorefinery concepts to achieve high efficiency levels. In this section, an integrated biorefinery system is devised based on the process technologies investigated in this work for the co-processing of wastes. The integrated co-processing of different waste streams in a biorefinery fashion has shown potential for improved economics and also as a technological solution towards a circular economy.^36^

**Fig. 8:**
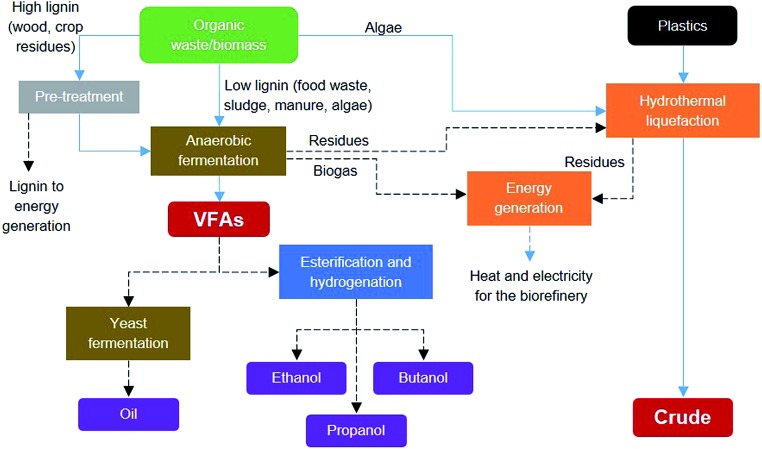
System integration for a waste biorefinery concept combining biochemical and thermochemical processes to produce platforms for biofuels or chemical production.

Fig. 8 shows the system integration for a waste biorefinery concept combining the processes investigated in this paper to produce platforms for biofuels or chemical production. Such an integrated system provides feedstock flexibility, allowing the ability to use any organic waste or biomass as the input and even co-process plastic wastes. Low lignin waste and de-lignified waste can be processed by anaerobic fermentation to produce short chain volatile fatty acids, as shown in Section 2.1. Although the HTL process was investigated for algae biomass and plastic waste in Section 2.2, this process can also utilize any solid residual streams from the organic waste processing, thus yielding more bio-crude product. Furthermore, solid residues from the HTL process can be used together with biogas from the anaerobic fermentation process for energy generation and to supply heat and electricity for operating the core biorefinery processes. This systematic integration of biochemical and thermochemical processing routes can potentially increase the carbon and energy recovery efficiencies while reducing residual streams and emissions. These efficiency gains are especially important for the sustainability of biorefineries.

**Fig. 9:**
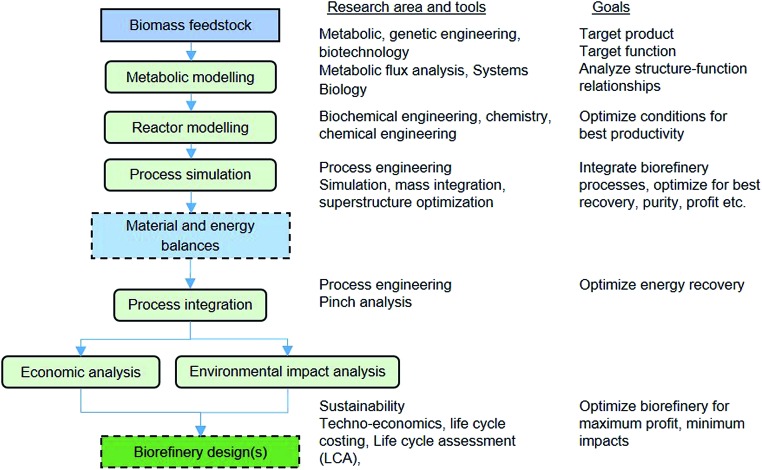
Systematic framework for process design and integration for an organic waste biorefinery.

Fig. 8 also shows the integration of alternative VFA conversions into other products. Biochemical processing by yeast can produce a palm oil substitute, which can be sold for cosmetics or to the food industry or for biodiesel production. The chemical synthesis route involves esterification and hydrogenation to produce mixed alcohols including ethanol, propanol and butanol, which can be sold as biofuels or as platform chemicals. The chemical synthesis route would require finding a way to supply the hydrogen required. Thus, another integrated route would be needed, possibly *via* anaerobic fermentation, which could be tuned to produce VFAs and hydrogen, or *via* steam reforming of biogas or gasification of solid residues. The selection of the best integration alternatives for a sustainable waste biorefinery would require extensive analysis and optimisation at all levels. Fig. 9 shows a systematic framework for biorefinery process design and integration. The figure also shows the goals at each level and the research areas and tools needed for a truly multidisciplinary approach to accelerate the development of sustainable biorefinery systems. Once the nature of the feedstock(s) is defined, the various steps in the framework involve the following:

#### 2.3.1. Metabolic modelling

With the advancement of metabolic and genetic engineering as well as computational capabilities, starting with the microorganism cells as the core of a biochemical process, as opposed to the reactor vessel, is now possible. To this end, metabolic engineering has largely contributed to mathematical modelling of biochemical reaction networks.^37^ Systems biology is advancing in terms of obtaining knowledge about the behaviour of microbial communities and their structure and function. Therefore it is now possible to understand the relation between microbial metabolisms, culture conditions and productivity to optimise microbial production in a biorefinery.^38^ This will allow synergistic tuning of mixed cultures such as in the anaerobic fermentation process shown in this paper.

#### 2.3.2. Process system simulation

VFAs are generally obtained in the reaction effluent in a diluted form together with other by-products, water and unreacted biomass. To obtain a marketable product, an appropriate combination of separation units (for filtration or centrifugation, or flash separators, distillation columns or liquid–liquid extraction columns, among others) is required. Process models are used in simulations to analyse the performance of the process integrated as a whole (reaction and separations) so that it can be optimised. The resulting mass and energy balance values are the basis for the next levels of analysis and optimisation.

#### 2.3.3. Process integration

It is important that energy and material inputs are used as efficiently as possible to offset fossil energy needs and greenhouse emissions. This is a standard step in chemical process design and pinch analysis can be employed to target energy recovery and make the biorefinery more energy efficient. Another important aspect is integration of the various processes in the biorefinery through material stream exchanges.^39^ For example, some processes can be sources of CO_2_ (*e.g.* fermentation) and others can be sinks of CO_2_ (*e.g.* algae cultivation) thus balancing the sources and sinks can provide higher efficiency levels.

#### 2.3.4. Economic analysis

This is a well-established step towards selecting a process design by evaluating economic performance indicators such as the payback, net present value, minimum selling price and economic margin potential. Traditional life cycle costing as well as a value analysis method for economic margin analysis of biorefinery processes could be used.

#### 2.3.5. Environmental impact analysis

An environmental impact analysis is needed to select the process alternatives that have the lowest potential for causing damage to the environment, ecosystems and human health. Traditional tools include carbon footprint and water footprint analyses and the more holistic life cycle assessment (LCA). Trade-offs between the environmental and economic objectives of a biorefinery system may arise during the analyses, thus to support decision making a simultaneous economic value and environmental impact (EVEI) analysis can be used.^39^

The technologies studied in this paper have the potential to enable high productivity and conversion of waste to valuable products in a biorefinery conceptualised through system integration and through employing a holistic multidisciplinary approach for system optimisation. This approach could open new possibilities for biorefining waste to produce biofuels and chemicals in a sustainable manner.

## 3. Experimental

### 3.1. Biological anaerobic conversion

#### 3.1.1. Substrates and inoculum

The inoculum from full scale anaerobic digesters (3.14 g TS L^–1^; 2.19 g VS L^–1^; 33 g COD L^–1^) and the food and liquid waste (Table 2) were supplied by GENeco (Wessex Water, UK).

**Table 2:** Physico-chemical characteristics of the municipal organic waste

	pH	COD_TOT_ g COD L^–1^	Sugars g COD L^–1^	Lactate g COD L^–1^	Ethanol g COD L^–1^	VFA g COD L^–1^	TS%	VS%
FW	4.6	157 ± 11	7.80	11.14	7.65	8.78	10.48 ± 0.03	9.56 ± 0.06
LW1	4.2	45 ± 5	0.03	2.48	35.91	2.16	1.09 ± 0.01	0.86 ± 0.01
LW2	7.5	7 ± 2	0.01	0.00	0.00	0.04	1.82 ± 0.01	0.69 ± 0.01
LW3	12.6	294 ± 1	0.63	0.44	0.00	2.82	12.99 ± 0.23	12.42 ± 0.24
LW4	6.2	87 ± 14	0.00	0.00	0.00	2.82	3.58 ± 0.01	2.91 ± 0.06
LW5	2.7	196 ± 24	78.66	1.26	0.00	0.00	17.47 ± 2.39	15.56 ± 4.81
LW6	2.7	114 ± 1	87.68	0.00	0.00	0.00	8.32 ± 0.07	7.68 ± 0.26

#### 3.1.2. Acidification potential (AP) and biochemical methane potential (BMP) tests

Batch experiments were carried out in 500 mL Schott® bottles immersed in a water bath at 35 °C. The bottles were topped with a rubber stopper containing three ports; one of them was connected to an automatic biogas counter (Bioprocess control AMPTS II), the second port was used for sampling and the third contained the vertical stirrer controlled by the system. The required amounts of substrate and inoculum were added according to the desired proportions (F/M) and the COD and VS concentrations, and topped up with tap water until a working volume of 400 mL was reached. The pH was initially adjusted to neutrality (7–7.5) with 2 M HCl or NaOH. For the AP tests, various F/M ratios and controls without the substrate were tested in triplicate over 7 days, sampling at days 1, 2, 4 and 7 of the experiment. The optimised AP tests consisted of F/M = 5 with 5 g COD L^–1^ of substrate and 1 g VS L^–1^ of inoculum. The degree of acidification was calculated according to Bengtsson *et al.*^40^ and the VFA yields according to Scoma *et al.*^17^ For the BMP tests, fixed amounts of 5 g COD L^–1^ of the substrate and 10 g VS L^–1^ of the inoculum (F/M = 0.5) were used for each substrate in triplicate, including controls for the inoculum. The tests were carried out over 30 days and sampling was performed at the end of the experiment. Specific Methane Production (SMP) and the kinetic parameters were calculated according to Angelidaki *et al.*^30^

### 3.2. Hydrothermal liquefaction (HTL)

#### 3.2.1. Substrate

*Ulva lactuca* Linnaeus (Ulva) was collected from Xom Con, Nha Trang, Khanh Hoa province, Vietnam on June 10, 2016. Prior to analysis and conversion, the macroalga was freeze-dried and milled to <1400 μm in diameter. The Spirulina *platensis* strain, classified as *Arthrospira* (Spirulina) *platensis*, was obtained from Hidumi Pharma Green Science Joint – Stock company, Vietnam, and used without subsequent purification.

#### 3.2.2. Reactors

Batch bomb-type reactors were fabricated according to literature precedent using stainless steel Swagelok® tube fittings.^41^ The reactor body consisted of a length of 1/2′′ tubing capped at one end, and connected at the other to a pressure gauge, thermocouple, and needle valve. The total internal volume of the reactors was *ca.* 9 mL.

#### 3.2.3. HTL procedure

An adapted reaction procedure, based on previous studies, was followed.^24^ In a typical reaction, the reactor was loaded with 0.5 g of the total solids (made up of biomass and 0–20% plastics) and 5 mL of freshly deionized water. The reactor was pressurised to 30 bar with compressed air and heated within a vertical tubular furnace set to 400 °C until the specified reaction temperature was reached (310 °C, ±10 °C, 60 min), and then removed from the furnace and allowed to cool to room temperature.

After cooling, the pressure was released *via* the needle valve. Following this, the aqueous phase was decanted from the reactor contents and filtered through a filter paper pre-dried overnight at 60 °C. The product yield in the water phase was determined by leaving a 2.5 mL aliquot to dry in a 60 °C oven overnight, and scaling the residue yield to the total aqueous phase mass.

To separate the remaining bio-crude oil and char phase, the reactor was washed repeatedly using chloroform until the solvent ran clear, which was then filtered through the same filter paper used to separate the aqueous phase (after drying for a minimum of 1 h). The filter paper and collected char were washed thoroughly with chloroform to remove all remaining bio-crude. The filtrate was collected and the solvent removed *in vacuo*. The char yield was calculated from the mass of the retentate collected on the filter paper after drying overnight in an oven at 60 °C.

Three repeat HTL runs using Spirulina with no additional plastic were carried out to determine the standard deviation in the mass balance values under the conditions examined.

### 3.3. Analysis

Total and volatile solid values were determined using gravimetric standard 2540G.^42^ The total and soluble COD were analysed using colorimetric kits (LCK014, Hach Lange®). HPLC (1260 Infinity, Agilent) was used to determine the sugars, VFAs and ethanol present with an Aminex® HPX-87H column (Bio-Rad) and a refractive index detector, using 5 mM H_2_SO_4_ as the mobile phase at 35 °C and 0.6 mL min^–1^. GC (7890A, Agilent) was used with an HP-PLOT/Q column at 35 °C for 5 min using He as the carrier gas, and FID for CH_4_ and TCD for CO_2_.

Elemental analysis was carried out externally at London Metropolitan University using a Carlo Erba Flash 2000 Elemental Analyser to determine the CHN content (elemental analyses were carried out at least in duplicate for each sample, and average values are reported).

Analysis of the biocrude was carried out using ^1^H NMR spectroscopy and GC-MS. ^1^H NMR spectroscopic measurements were carried out at 298 K using a Bruker AV400 spectrometer, operating at 400 MHz for ^1^H. Typically, the samples were analyzed in CDCl_3_ and the spectra were referenced to the residual CHCl_3_ peak from the solvent (*δ* 7.26 ppm). GC-MS analysis was carried out using an Agilent 7890A Gas Chromatograph equipped with a CP-Sil capillary column (25 m × 0.250 mm internal diameter) and a He mobile phase (flow rate: 1.2 mL min^–1^), coupled with an Agilent 5975C MSD. Approximately 50 mg of each sample was dissolved in 100 mL of hexane and 1 μL of each solution was loaded onto the column, pre-heated to 40 °C. This temperature was held for 1 minute, and then the temperature was increased to 250 °C at a rate of 10 °C min^–1^ and then held for 10 minutes.

^14^C analysis was performed by Beta Analytic Inc. (Florida, USA) according to ISO/IEC 17025:2005.

## 4. Conclusions

Organic waste valorisation with complex and variable compositions is possible using anaerobic fermentation processes and/or HTL processes. To unravel the chemical and energy potentials during biological conversions, assessment of the substrate using BMP or AP tests is required, and the latter were optimised using a F/M ratio of 5 to allow all the chemical conversions to occur. Feedstocks presenting high hydrolysis kinetics are recommended to be used in fermentation instead of AD for recovering chemicals with added value. This study has also demonstrated that it is feasible to use HTL to convert opportunistic algal biomass as a feedstock, and that not only is there no need to separate out plastic detritus from the organic matrix prior to processing but the plastic itself could improve the economic viability of the process. System integration and a holistic multidisciplinary approach could open new possibilities for biorefining waste to produce biofuels and chemicals in a sustainable manner.
